# Detection of Bioactive Metabolites in *Escherichia Coli* Cultures Using Surface-Enhanced Raman Spectroscopy

**DOI:** 10.1177/00037028221079661

**Published:** 2022-03-25

**Authors:** Heera Jayan, Hongbin Pu, Da-Wen Sun

**Affiliations:** 1 School of Food Science and Engineering, 26467 South China University of Technology, Guangzhou, China; 2 Academy of Contemporary Food Engineering, South China University of Technology, Guangzhou Higher Education Mega Center, Guangzhou, China; 3 Engineering and Technological Research Centre of Guangdong Province on Intelligent Sensing and Process Control of Cold Chain Foods, & Guangdong Province Engineering Laboratory for Intelligent Cold Chain Logistics Equipment for Agricultural Products, Guangzhou Higher Education Mega Centre, Guangzhou, China; 4 Food Refrigeration and Computerized Food Technology (FRCFT), Agriculture and Food Science Centre, 8797 University College Dublin, National University of Ireland, Dublin, Ireland

**Keywords:** *Escherichia coli*, indole production, surface-enhanced Raman spectroscopy detection, SERS detection, metabolic products

## Abstract

Detection of bioactive metabolites produced by bacteria is important for identifying biomarkers for infectious diseases. In this study, a surface-enhanced Raman spectroscopy (SERS)-based technique was developed for the detection of bioactive metabolite indole produced by *Escherichia coli* (*E. coli*) in biological media. The use of highly sensitive Au@Ag core-shell nanoparticles resulted in the detection of indole concentration as low as 0.0886 mM in standard solution. The supplementation of growth media with 5 mM of exogenous tryptophan resulted in the production of a maximum yield of indole of 3.139 mM by E. coli O157:H7 at 37 °C. The growth of bacterial cells was reduced from 47.73 × 10^8^ to 1.033 × 10^6^ CFU/mL when the cells were grown in 0 and 10 mM exogenous tryptophan, respectively. The amount of indole in the Luria–Bertani (LB) media had an inverse correlation with the growth of cells, which resulted in a three-log reduction in the colony-forming unit when the indole concentration in the media was 20 times higher than normal. This work demonstrates that SERS is an effective and highly sensitive method for rapid detection of bioactive metabolites in biological matrix.

## Graphical abstract

**Graphical Abstract:**
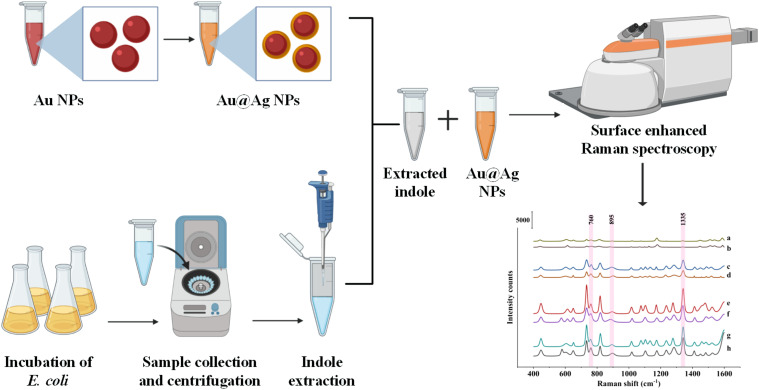

## Introduction

Microorganisms play a significant role in human life and they are found in the environment, food, and even in the intestinal tract of animals. Several bacterial species produce bioactive metabolites as a surviving method to acquire adaptability to adverse environmental conditions and resistance against stressors Microorganism plays a significant role in human life and they are found in the environment, food, and even in the intestinal tract of animals.^1,2^ Several bacterial species produce bioactive metabolites as a surviving method to acquire adaptability to adverse environmental conditions and resistance against stressors.^3^ These metabolites perform several functions during the growth of the cell including intercellular signaling compounds, toxins, and virulence factors.^4,5^ These bioactive metabolites take part in modulating virulence properties of the bacterial species and thus affect human health.^6,7^

*Escherichia coli* (*E. coli*) is a gram-negative, facultative anaerobic, and rod-shaped bacterium that has been associated with various urinary tract infections, hemorrhagic colitis, and hemolytic uremic syndrome in humans.^8^ *E. coli* produces indole that acts as a signaling compound to regulate genes responsible for various physiological processes including stress responses,^9^ drug resistance,^10^ biofilm formation,^11^ and persister cell development.^12^

In bacterial cell tryptophanase, a cytoplasmic enzyme hydrolyzes tryptophan and produces indole pyruvate and ammonia. Tryptophanase is encoded by the *tnaA* gene in the *tnaCAB* operon, in which *tnaB* enables transportation of tryptophan across the cell membrane. Catabolite repression plays an important role in the transcription of tryptophanase gene.^13^ The amount of tryptophan decides the transcriptional attenuation, which means that when tryptophan levels are low, premature termination of transcription occurs, creating repression in tryptophanase expression and vice versa. The tna operons regulate the extracellular indole production in *E. coli* and the gene expression of *tnaAB* is affected by several factors including carbon source,^14^ temperature,^15^ pH,^16^ and the presence of other microbial species.^17,18^

Recent studies have shown that indole has the ability to regulate the expression of *tnaB*, *astD*, and *gabT* in *E. coli* planktonic cells.^19^ Indole is also known to be associated with control of virulence, Shiga toxins production,^20^ biofilm formation, flagella production, motility,^21^ chemotaxis,^22^ and plasmid multimerization^23^ in the bacterial cell. Several indole analogs have been found to naturally occur in the environment. The chemical modification of indole can occur in indole producing or non-indole producing bacterial cells and the commonly found indole derivatives include 3-methylindole, indole-3-propionic acid, and indole-3-carbinol.^24^ Thus, the detection of indole production by enteric bacteria is an important phenotypic characteristic that can be used to differentiate bacterial cells and identify bacterial infections.^25^

The most commonly employed technique for the detection of indole is Kovac’s assay,^16^ which is based on the ability of p-dimethylaminocinnamaldehyde to form a red dye by reacting with indole. However, Kovac’s reagent tends to react with all indole containing compounds and this nonspecific reaction limits the application of Kovac’s assay for quantitative detection.^26^ On the other hand, chromatographic techniques such as high-performance liquid chromatography and gas chromatography–mass spectrometry (GC–MS) have been proposed for highly specific detection in complex biological media,^27^ however, these expensive and labor-intensive techniques require long processing time; therefore, it is necessary to develop a cheap, simple, and rapid method for the detection of metabolic products from complex biological systems.^28,29^

Surface-enhanced Raman spectroscopy (SERS) is an excellent tool for providing the molecular fingerprint of the components present in samples and has been used for quantitative detection of bioactive metabolites produced by microorganisms.^30^ Surface-enhanced Raman spectroscopy is an excellent tool for providing the molecular fingerprint of the components present in samples and has been used for quantitative detection of bioactive metabolites produced by microorganisms.^30–32^

De Marchi et al.^33^ employed SERS to analyze the spatial distribution of bioactive metabolites produced by mixed culture of *E. coli* and *Pseudomonas aeruginosa* colonies on agar media, but the study did not include quantitative detection, while Dieng and Schelvis^34^ predicted the isotope shift of Raman bands in various isotopomers of indole in an attempt to provide proper assignment and interpretation of Raman bands in the spectrum. In addition, Gaimster et al.^35^ showed that the quantification of indole in the growing culture of *E. coli* mainly depended on Kovac’s assay.

However, the above studies only examined the diverse effect of indole on various phenotypic characteristics of the bacterial cells,^16^ and little information is available on the maximum amount of indole bacterial cells that can produce and the factors that determine the final yield. Therefore, in the current study, indole produced by wild-type *E. coli* O157:H7 was investigated using surface-enhanced Raman spectroscopy with Au@Ag core-shell nanoparticles as the substrate, and its effects on cell growth were analyzed. The selected substrate of Au@Ag nanoparticles constituting gold nanoparticles (core) encapsulated in Ag nanoparticles (shell) exhibited a higher SERS enhancement capability than Au nanoparticles alone under excitation. It is hoped that the proposed method could be applied to other microbial communities and mixed cultures. To the best of our knowledge, this is the first quantitative analysis of indole production by *E. coli* cells using SERS.

## Materials and Methods

### Materials

Tetrachloroauric(III) acid trihydrate (HAuCl_4_·3H_2_O) and rhodamine 6G (C_28_H_31_ClN_2_O_3_) were purchased from Aladdin Reagent Co., Ltd. (Shanghai, China). Trisodium citrate dihydrate (C_6_H_9_Na_3_O_9_), ascorbic acid (C_6_H_8_O_6_), indole (C_8_H_7_N), L-tryptophan (C_11_H_12_N_2_O_2_), iso-amyl alcohol (C_5_H_12_O), and ethyl acetate (C_4_H_8_O_2_) were supplied by Shanghai Macklin Biochemical Co., Ltd. (China). Luria–Bertani (or lysogeny broth, LB) broth and LB agar were acquired from Guandong Huankai Microbial Sci. and Tech. Co., Ltd. (Guangzhou, China). Para-di-methyl amino benzaldehyde (C_9_H_11_NO), hydrochloric acid (HCl), silver nitrate (AgNO_3_), and sodium hydroxide (NaOH) were bought from Sinopharm Chemical Reagent Co., Ltd. (Beijing, China). Deionized water was used for the synthesis of nanoparticles and distilled water for culture media preparation.

### Synthesis and Characterization of Core-Shell Nanoparticles

Citrate stabilized gold nanoparticles were prepared based on the method previously reported by Hussain and team.^36^ In brief, 1 mL of HAuCl_4_.3H_2_O (5 g/L) was added into 60 mL of boiling deionized water, followed by the addition of 700 µL of trisodium citrate (1%) under vigorous stirring by a magnetic stirrer (MS7-H550-Pro, DLAB Scientific Co., Ltd., Beijing, China). The mixture was allowed to stir for 15 min for the formation of gold nanoparticles as indicated by the change of color to wine red, which was then stored at 4 °C until further analysis.^37–39^ The silver coating on gold nanoparticles was achieved by the previously reported method.^40–42^ An amount of 3 mL of colloidal gold nanoparticles was centrifuged (JW-3024HR, Anhui Jiaven Equipment Industry Co., Ltd., Hefei, China) and redispersed in 3 mL of water and kept on a digital shaker (MS 3, IKA Inc., Staufen im Breisgau, Germany) at 1000 rpm followed by the addition of 120 µL of ascorbic acid (10 mM). Then, a dropwise addition of 120 µL of silver nitrate (10 mM) was introduced into the mixture and the reaction was allowed to continue for 30 min. The formation of the silver layer on the surface of the gold nanoparticles was indicated by the change of color from wine red to orange. The particle was washed and redispersed in water using the centrifuge, which was then stored at 4 °C until further use.

The prepared gold core and Au@Ag core-shell nanoparticles were analyzed using a UV–visible (UV–Vis) spectrophotometer (UV-1800, Shimadzu Co., Kyoto, Japan), and the surface morphology was analyzed using a high-resolution transmission electron microscope (TEM) (JEM-2100F, Jeol Ltd., Tokyo, Japan) operating at 200 kV. The particle size distribution was analyzed using Zetasizer Nano ZS (Malvern Panalytical Ltd., Malvern, UK). The enhancement ability and sensitivity of the nanoparticles were analyzed using a confocal Raman spectroscope (LabRAM HR, Horiba France SAS, Villeneuve d'Ascq, France) with rhodamine 6G (R6G) at different concentrations (10^–5^ to 10^–10^ M) as a probe molecule.

### Bacterial Strain and Culturing Methods

The *E. coli* O157:H7 (ATCC 25922) used for this study was provided by Guangzhou Microbial Culture Centre (Guangzhou, China) and LB broth was used as the medium for growth. The bacterial strains were cultured from –80 °C glycerol stock in LB broth and incubated at 250 rpm and 37 ± 0.5 °C overnight in an incubator (SPX-150, Zhong Yi Guo Ke (Beijing) Technology Co., Ltd., Beijing, China). The overnight cultures were diluted with LB broth to reach the optical density (OD) of 0.2 at 600 nm and the culture was supplemented with 0, 1, 5, and 10 mM of tryptophan. The culture was incubated at 37 °C for 24 h with gentle agitation and samples were collected at different time intervals. The growth rate was assessed by plotting absorption OD_600_ versus different time intervals. The number of cells in the medium at the end of the incubation period was enumerated using LB agar plate and represented as colony-forming units (CFU)/mL.^16,43^

### Indole Assays

The production of indole by bacteria was analyzed using SERS with core-shell nanoparticles as a substrate. An amount of 1 mL of bacterial culture was collected at different time intervals and centrifuged at 10 000 rpm for 10 min at 4 °C and the supernatant was collected and stored at 4 °C until further use. The indole present in the supernatant was extracted two times using the ethyl acetate and the solvent was completely evaporated at 50 °C in a hot air oven (Shanghai Yiheng Instruments Co., Ltd., Shanghai, China). The remaining content was redispersed in water and used for Raman spectroscopic analysis as mentioned above. For comparison, the indole was also quantified using Kovac’s assay. Kovac’s reagent was prepared by mixing 5 g of para-di-methyl amino benzaldehyde to the mixture of iso-amyl alcohol (25 mL) and hydrochloric acid (75 mL). Further, 150 µL of Kovac’s reagent was added to 100 µL of supernatant and the mixture was incubated for 30 min, then the upper layer was diluted to 1:10 in HCl–amyl alcohol solution (30 mL of HCl and 90 mL of amyl alcohol). The absorbance of the resulted mixture was measured at 530 nm using the UV–Vis spectrophotometer.

### Surface-Enhances Raman Spectroscopy Methods

The spectroscopic analysis was conducted using SERS. The spectroscopy system was equipped with a confocal microscope, a flat-field achromatic monochromator with a focal length of 800 mm, and a charge-coupled device detector. The extracted indole and the substrate were mixed in a 1:1 ratio and mounted on to confocal Raman spectroscope in a glass capillary tube (inner diameter: 1 mm), and the excitation was carried out at 532 nm (50 mW). The spectra were collected using a 10x objective and a 600 grooves/mm diffraction grating. The accumulation time was set as 10 s for each of the four scans acquired. A linear standard curve was obtained by plotting the concentration of indole in standard solution (0.05–10 mM) against the corresponding Raman intensity obtained. The limit of detection was calculated as follows:

(1) $$LOD=\;3S_{b}+\;Y_{b}$$

where S_b_ is the standard deviations of the blank sample and Y_b_ is the average relative intensity of the blank sample.

### Statistical Analysis

All the experiments conducted in this study were performed in triplicate, results obtained were represented as mean ± standard deviation and all the graphs were plotted using OriginPro 2018 (OriginLab Co., Northampton, Massachusetts, USA). The diameter of the nanoparticles was obtained from TEM images in their original magnification using ImageJ software (National Institutes of Health, USA). Raman spectra were processed using LabSpec6 software (Horiba France SAS, Villeneuve d'Ascq, France) for despike processing, smoothing, and baseline correction.

## Results and Discussion

### Characterization of Core-Shell Nanoparticles

Gold nanoparticles with highly uniform core size were selected as the core and silver as the shell to achieve high enhancement capability.^44^ The reduction of Ag^+^ ions on citrate stabilized gold nanoparticles was obtained by stepwise addition of silver nitrate in the presence of a mild reducing agent, while preventing the formation of free Ag nuclei.^45,46^

**Figure 1.**
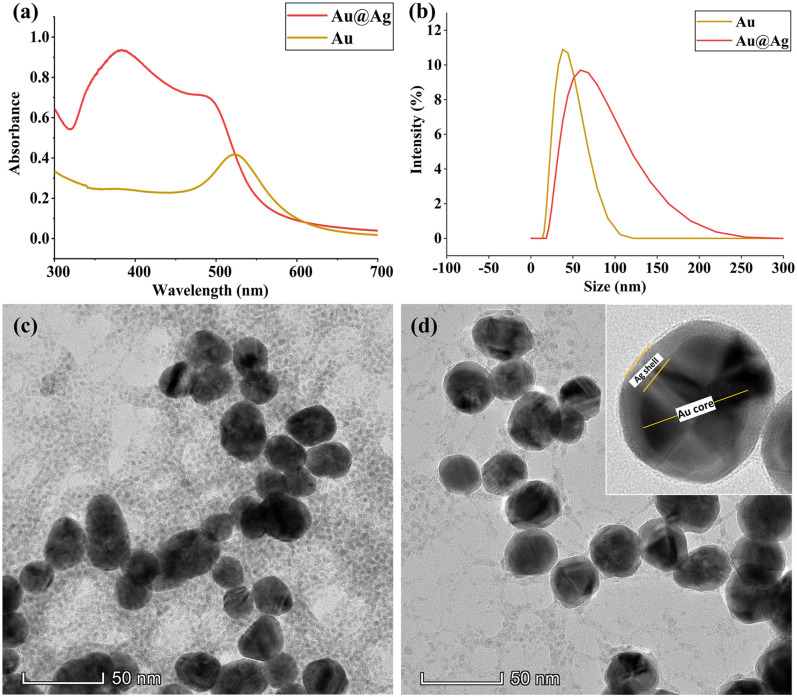
(a) UV–Vis spectra of gold core and core-shell nanoparticles. (b) Particle size distribution of gold and core-shell nanoparticles and high-resolution TEM images of (c) gold and (d) core-shell nanoparticles (Inset: Single Au@Ag core-shell nanoparticle).

The surface plasmon resonance was measured using ultraviolet–visible (UV–Vis) spectroscopy (Fig. 1a). The core-shell nanoparticles showed two distinctive absorption peaks, which corresponded to silver and gold at 380 and 489 nm, respectively. It is also interesting to note that the localized surface plasmon resonance (LSPR) of the gold in the core-shell nanoparticles was blueshifted from that of the LSPR of pure gold colloids (524 nm). The double peak spectra of Au@Ag core-shell nanoparticles and blueshift of gold peak indicated the synergistic optical features of bimetallic core-shell nanoparticles, which was not a simple physical mixture of two different kinds of metal nanoparticles.^47–49^ The particle size distribution of gold and Au@Ag nanoparticles was centered around 37.8 and 58.8 nm, respectively (Fig. 1b). The morphological characteristics of the synthesized nanoparticles revealed by TEM are shown in Fig. 1c and d. A uniform coating of silver on spherical gold nanoparticles resulted in quasi-spherical Au@Ag cores shell nanoparticles.^50,51^ The TEM image shows a clear distinction between Au core and Ag shell due to the difference in electron density of Au and Ag atoms (Fig. 1d, inset).

**Figure 2.**
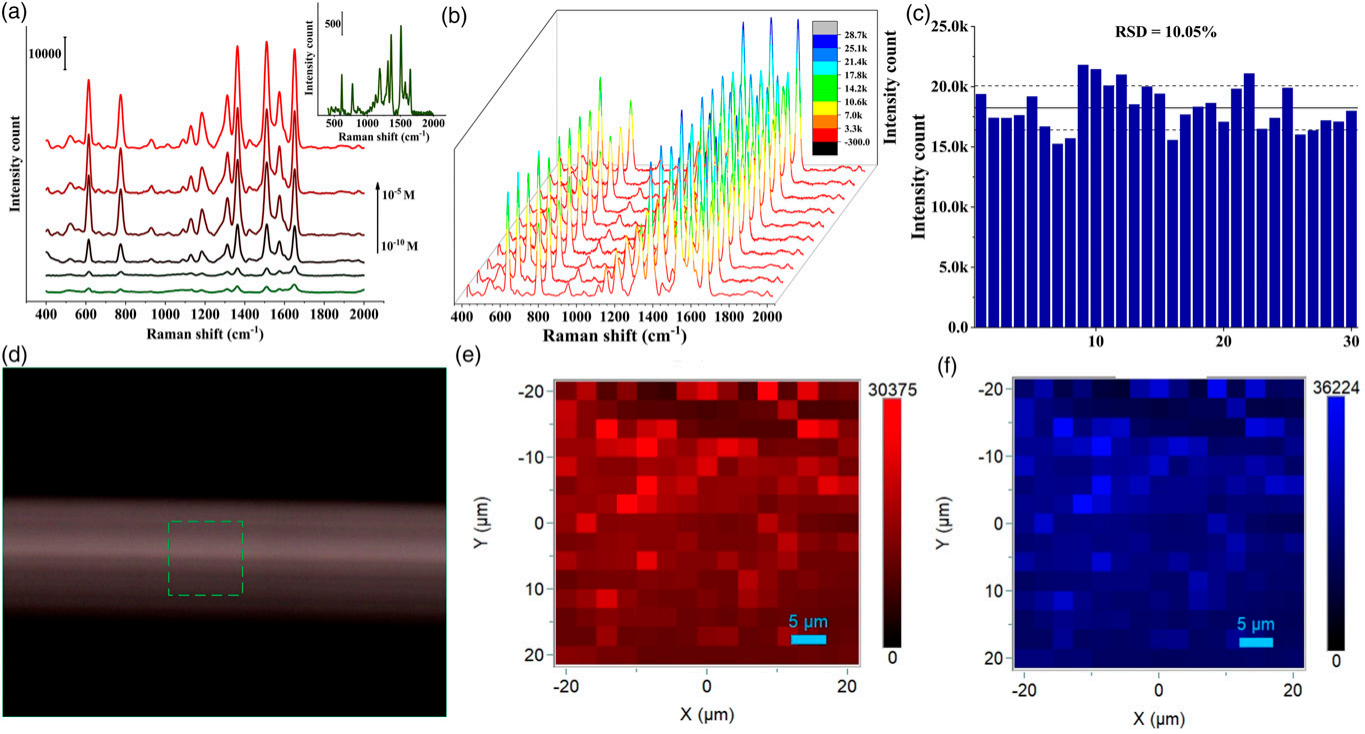
(a) SERS spectra of R6G at different concentrations from 1 × 10^−5^ to 1 × 10^–10^ M collected using Au@Ag nanoparticles, (b) SERS spectra of 1 × 10^–7^ M R6G collected at 10 random points, (c) relative standard deviation (RSD) of the specific Raman mode of 30 random points at 614 cm^−1^, (d) optical image of the area selected for mapping, Raman maps of 1 × 10^–7^ M R6G by targeting the Raman shift at (e) 614 cm^–1^, and (f) 1362 cm^–1^.

To explore the enhancement capabilities of the prepared substrate, the R6G molecule was used as the Raman probe molecule. The Raman spectra of varying concentrations of R6G molecules ranging from 10^–5^ to 10^–10^ M with the substrate are shown in Fig. 2a. The characteristic peaks for the Raman spectra were clearly visible at high concentrations, and with decreasing the concentration, the intensity also decreased. The characteristic peaks of R6G at 614 and 1310 cm^−1^ corresponded to the in-plane C–C–C bending and C–O–C bending, respectively, and the peaks at 771 and 1125 cm^−1^ could be assigned to C–H out-of-plane and in-plane bending modes.^52^ For comparison, the performance of the prepared substrate was calculated in terms of enhancement factor (EF) using the following formula:^53,54^

(2) $$EF=\frac{I_{SERS}}{C_{SERS}}x\;\frac{C_{Normal}}{I_{Normal}}$$

where I_SERS_ and I_Normal_ are the intensities of the characteristic peaks of R6G from SERS and normal Raman spectra and C_SERS_ and C_Normal_ are the corresponding concentrations, respectively. The EF for core-shell nanoparticles was calculated to be 3.7 × 10^6^, which was 200 times higher than gold nanoparticles (1.837 × 10^4^). The SERS spectra of 10^–7^ M R6G with gold nanoparticles are shown in Fig. 2a (inset). To validate the uniformity, Raman spectra were collected from 10 random spots on the sample containing substrate and R6G (10^–7^ M). The signal intensity was consistent for all characteristic peaks of R6G as shown in Fig. 2b. The relative standard deviation for the 30 spots was found to be 10.05 % at the peak of 614 cm^–1^, which indicated the high uniformity of SERS performance (Fig. 2c). This exhibited that the prepared core-shell nanoparticles were highly reliable and uniform, which is essential to generate reproducible Raman data for practical applications.^55,56^ In addition, the intensity distribution as a function of position was measured by obtaining 225 spectra across an area of 1600 µm^2^ (Fig. 2d). The SERS mapping image at the peak 614 and 1362 cm^–1^ is shown in Fig. 1e and Fig. 1f, respectively. The Raman maps showed uniform color and no drastic fluctuations in the intensity, which further ensured the uniformity and reproducibility of the substrate.

### Detection of Indole in Standard Solution

**Figure 3.**
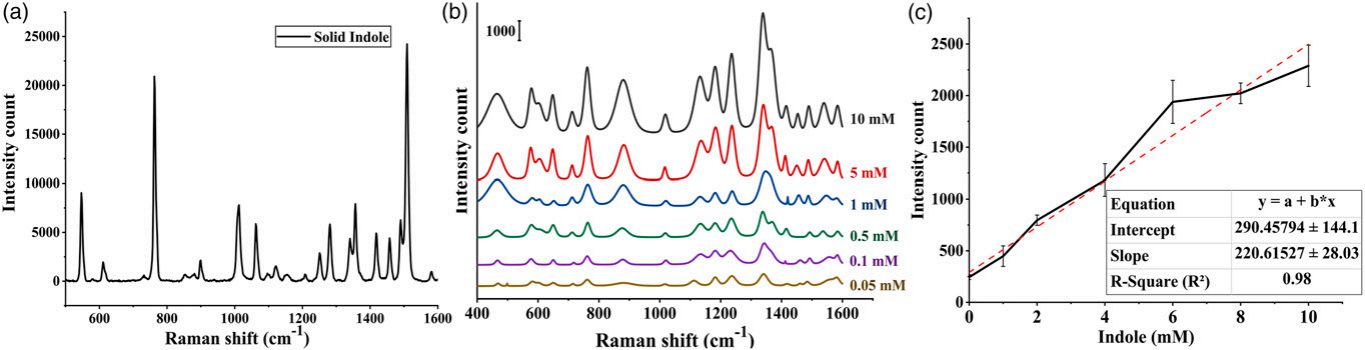
(a) Raman spectra of solid indole, (b) SERS spectra of standard indole solution at different concentrations ranging from 10 to 0.05 mM, and (c) calibration curve plot of indole SERS intensity at 760 cm^–1^ against different concentrations.

The vibrational spectroscopic features of the standard solution of indole were analyzed using Au@Ag core-shell nanoparticles. The spectra revealed several dominant peaks at 760, 878, 1010, 1335, 1415, and 1443 cm^–1^, which were in accordance with the Raman spectrum obtained from the solid indole (Fig. 3a). The two peaks at 760 and 1010 cm^–1^ were assigned to indole ring in-phase and out-of-phase breathing modes, respectively. Additionally, the Raman band at 878 cm^–1^ was assigned to the N–H bending in the indole ring. A comprehensive band assignment of Raman bands indole is shown in Table I.^33,57,58^ The N–H band in indole was a proton donor, which resulted in the formation of a hydrogen bond with negatively charged nanoparticles, causing the downward shift in Raman bands especially at the peak of 895 cm^–1^.^59,60^ These substantial downward shifts observed in our study suggested a strong interaction between the indole ring and the nanoparticles. This characteristic of the pyrrole ring was further confirmed in the study conducted by Chuang and Chen,^61^ who showed that there was no significant peak observed for the pyrrole ring when positively charged silver nanoparticles were used as the substrate.

**Table I.** Raman indole solutions.

Raman shift (cm^–1^)	Band assignment	Reference
600	C–C bending in benzene ring	33
760	Out-of-phase pyrrole ring breathing	57
895	Hydrogen bond interaction at NH site	58
1019	In-phase pyrrole ring breathing	57
1233	In-plane skeletal modes of the pyrrole ring	57
1335	Out-of-plane bending vibrations pyrrole ring	58
1415	In-plane pyrrole ring bending	57
1450, 1489	C–C stretching in benzene ring	33
1542	Pyrrole ring stretching vibration ν(C2=C3)	58
1587	Benzene e2θ band	57

The SERS spectra of the solution containing different concentrations of indole ranging from 10 mM to 0.05 mM were also analyzed using core-shell nanoparticles. Figure 3b shows the average spectrum for all concentrations of indole including 10, 5, 1, 0.5, 0.1, and 0.05 mM. It was observed that as the concentration decreased, the peak intensities at 895 and 760 cm^–1^ decreased gradually, and the peaks were still present at concentrations as low as 0.05 mM. Moreover, the peaks at 1010 and 1335 cm^–1^, respectively, representing in-phase breathing and N–H deformation in pyrrole ring were still observable in all the spectra.^57,58^ For quantitative detection of indole, the peak intensity at 760 cm^–1^ was plotted against the concentrations and the linear response was observed over the concentrations of 0–10 mM with a correlation coefficient (R^2^) of 0.98 (Figure 3c). The limit of detection was calculated to be 0.0886 mM, which was below the biologically relevant concentration of indole produced by *E. coli*.^13^

### Surface-Enhanced Raman Spectroscopy Analysis of Indole

**Figure 4.**
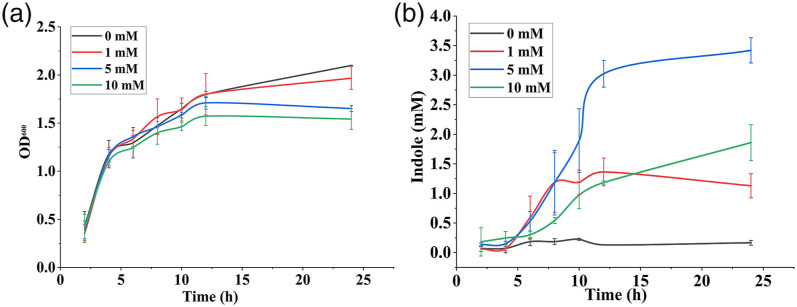
(a) Cell growth of *E. coli* as represented by the optical density measured at 600 nm at different time intervals and (b) extracellular indole production by *E. coli* during incubation at different time intervals obtained by Kovac’s assay.

**Figure 5.**
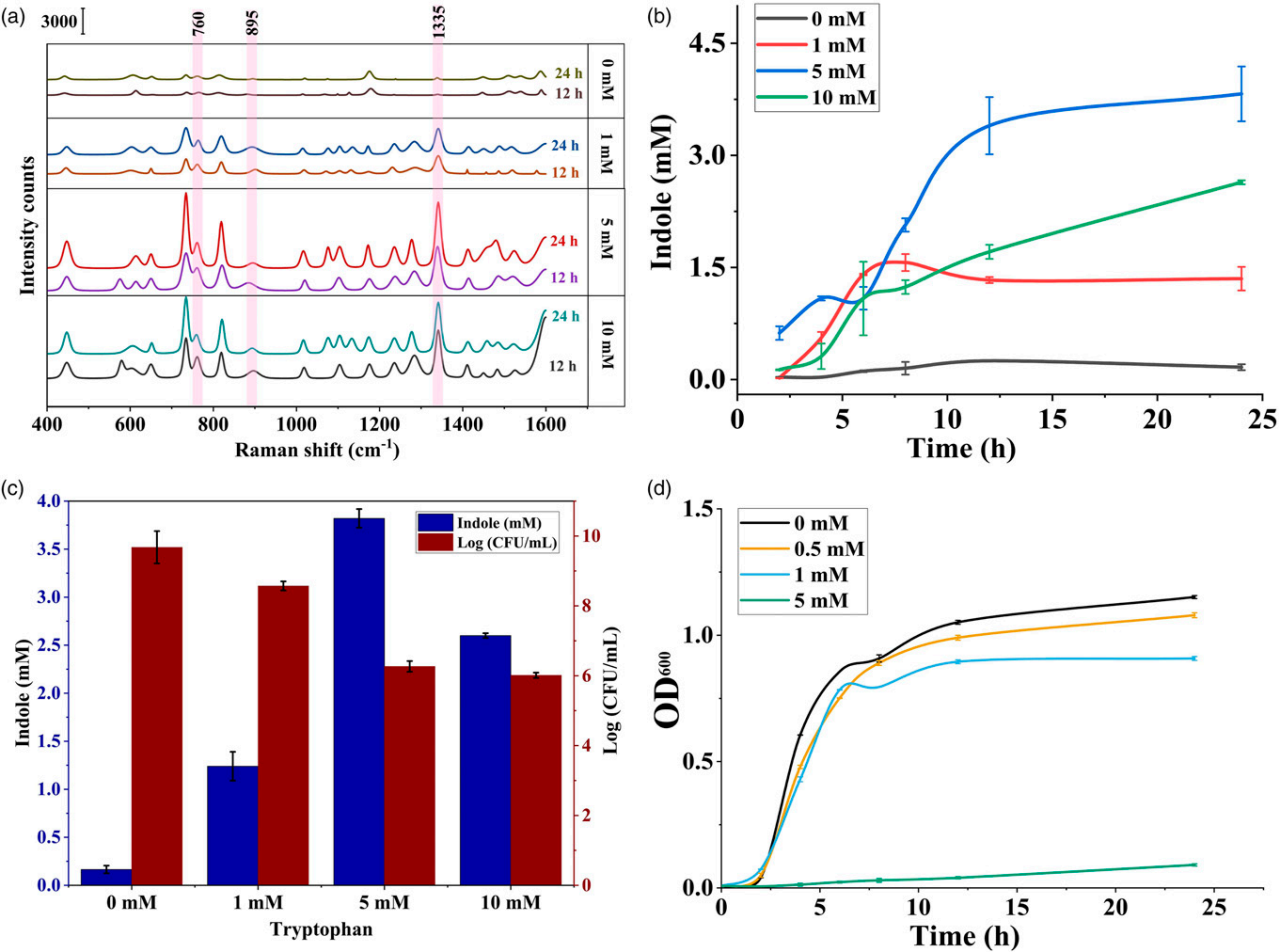
(a) SERS spectra of indole extracted from samples after incubation for 12 h and 24 h supplemented with 0, 1, 5, and 10 mM exogenous tryptophan, (b) indole concentration at different time intervals measured using SERS, (c) indole production obtained using the SERS method and *E. coli* cell growth after incubation for 24 h, and (d) cell growth of *E. coli* cultivated in different concentrations of exogenous indole (0, 0.5, 1, and 5 mM).

The indole produced by *E. coli* in the LB broth was extracted two times using ethyl acetate and the solvent was evaporated, and the content was redispersed in water, which was then used for Raman spectroscopic analysis with core-shell nanoparticles as the substrate at a 1:1 ratio. The obtained results were compared with the values obtained from Kovac’s assay (Figure 4b). The Raman peaks for all four samples (0, 1, 5, and 10 mM tryptophan) after incubation for 12 and 24 h are shown in Figure 5a. It is important to consider the fact that Raman spectra obtained from biological media often contain interference from background signals and contributions from multiple components that mask the characteristic peaks of the targeted metabolite. The spectra obtained from the extracted indole showed characteristic peaks at 760, 895, and 1335 cm^–1^, which was in accordance with the peaks obtained for the standard solution of indole. It is interesting to note that the extracted indole spectrum is almost similar to characteristic peaks of commercial indole. However, the spectra also contained two additional peaks at 730 and 820 cm^–1^, which might be caused by the presence of adenine and thymine in the media,^62^ but these peaks did not interfere with the characteristic peaks of indole and the SERS spectra differed from the standard indole spectra only in the two aforementioned peaks. Similar results were shown by De Marchi et al.,^33^ who detected indole produced by *E. coli* MG1655 strain grown on LB agar and comparison of different technique available for detection of indole is provided in Table II.^26,63,64^ Moreover, tryptophan has a similar molecular structure as indole, but tryptophan showed different characteristic peaks including 778, 914, and 1363 cm^–1^ than indole, thus confirming that the tryptophan was not an interfering compound during the detection of indole.^65^

**Table II.** Comparison of different methods available for detection of indole.

Technique	Limitation	Sample	LOD	Reference
Kovac’s assay	Nonspecific to other indole analogs	Culture media	0.1 µM	63
Hydroxylamine indole assay (HIA)	Background interference in absorbance	Fecal sample	—	26
Headspace solid-phase microextraction coupled to GC-MS	Extensive and labor-intensive sample pretreatment procedure	Culture media	0.06716 µM	64
SERS	Qualitative analysis	Agar plate	—	33
SERS	—	Culture media	0.0886 mM	Current study

The wild-type *E. coli* O157:H7 has an open reading frame homologous to *tnaA* in *E. coli* K-12, which encodes the production of tryptophanase enzyme.^66^ The concentration of indole produced by *E. coli* during incubation at 37 °C in the presence of exogenous tryptophan was analyzed. For *E. coli* cultured in LB broth without the presence of exogenous tryptophan, the extracellular indole concentration in the supernatant reached 0.1278 mM after incubation for 24 h, which was consistent with the fact that LB broth itself contains 0.5–0.6 mM of tryptophan. The accumulation of indole in supernatant increased consistently over time for all samples until it reached the stationary phase. This result was consistent with a previous study as reported by Han et al.^16^ who conducted an experiment on *E. coli* K-12 BW25113 and observed the indole production at 37 °C was ∼ 0.15 mM.

Figure 4 shows the production of indole at different time intervals obtained from Kovac’s assay and the corresponding cell growth measured in terms of optical density at 600 nm. The indole concentration at 5 mM exogenous tryptophan was increased to 2.07 mM indole/OD after incubation for 24 h, although the cell density of bacterial culture was 1.27 times lower than that of culture without tryptophan supplementation. The indole/OD of culture with 1 (0.5742 mM indole/OD) and 10 mM (1.206 mM indole/OD) of exogenous tryptophan was 7.3 and 15.3 times higher than that of the culture with 0 mM (0.07878 mM indole/OD) exogenous tryptophan. Similarly, Han et al.^16^ observed that the indole concentration in *E. coli* K-12 BW25113 cultivated at 37 °C was 79 ± 2 µM indole/cell OD at 14 h.

The indole concentration measured using the SERS method at different time intervals is shown in Fig. 5b. A gradual increase in indole concentration was observed to be similar to the results obtained using Kovac’s assay. The final indole concentration in the media obtained using SERS is shown in Fig. 5c. The indole concentration in the bacterial culture increased based on the amount of exogenous tryptophan available in the medium in a dose-dependent manner. Thus, *E. coli* was incubated in the presence of 1, 5, and 10 mM of tryptophan under the same environmental conditions and to the same extent. In the presence of 1mM tryptophan the extracellular indole concentration reached up to 1.24 ± 0.15 mM, which indicated that the bacterial cell was able to convert most or all exogenous tryptophan to indole. In the presence of 5 mM of tryptophan, the indole concentration reached 3.82 ± 0.09 mM after incubation for 24 h. However, at a very high concentration of exogenous tryptophan, the cells were able to convert only a small portion of tryptophan, reaching a final indole concentration of 2.6 ± 0.03 mM. This reduction in the concentration of indole indicated that the maximum amount of extracellular indole *E. coli* O157:H7 under the same environmental condition that could produce was approximately 3–4 mM. This was consistent with the results obtained by Li and Young^13^ who reported that the final concentration of indole produced by *E. coli* solely depended on the amount of exogenous tryptophan available and the highest concentration of indole production was found to be 5 mM. Furthermore, as the exogenous tryptophan was exhausted, the extracellular indole accumulation slowed down.

### Effects of Indole on Cell Growth

The effect of indole on cell growth was analyzed by the enumeration of *E. coli* cells after incubation for 24 h on LB agar. The cells grown in LB broth supplemented with different concentrations of tryptophan were enumerated and compared with the final amount of indole present in the media and the results are shown in Fig. 5c. It is evidently clear that with an increase in the accumulation of indole in the media, the CFU/mL of *E. coli* gradually decreased. This indicated that the increase in indole production resulted in reduced cell growth and development. The bacterial population in LB media with 1 mM exogenous tryptophan was found to be 37.33 × 10^7^ CFU/mL, which was 12 times lower as compared with the control sample (47.73 × 10^8^ CFU/mL). The transportation of tryptophan into the cell was carried out by tryptophan-specific transporter *tnaB*, promoting sufficient concentration to induce *tnaA* expression. The tryptophan transport was not triggered by the initiation of the *tnaA*, indicating that the bacterial cell growth was unaffected by the presence of *tnaA* in absence of exogenous tryptophan.^13^

Previous studies have shown that the production of indole in cells was not uniform throughout the cell growth, but a rapid accumulation of indole took place during the period of transition from exponential to stationary phase.^67^ During this period, if the concentration of exogenous tryptophan is as high as 10 mM, indole is produced faster than it can be transferred out of the cell, which results in a rapid rise in cell-associated indole. This phenomenon is termed as indole pulse and the amount of intracellular indole at this point is thought to be raised to 60 mM, which is a very high concentration that can only be obtained by the addition of 4 mM of exogenous indole to the medium.^35^ This indole pulse inhibits cell growth and cell division.^68^ Indole reduces the electrical potential difference across the cell membrane by acting as a proton ionophore and prevents Z ring formation during cell division by inhibiting the MinCD system.^69^ The combined effect of cell growth and division inhibition by the high intracellular concentration of indole explains the reduction in the colony from 47.73 × 10^8^ to 1.033 × 10^6^, which were grown in the presence of 0 and 10 mM exogenous tryptophan, respectively. A similar observation was made by Gaimster et al.^70^ who compared the long-term stationary phase viability of *E. coli* BW25113 and its complementary mutant (BW25113 Δ*tnaA*) that cannot produce indole with or without the presence of indole. Their results suggested that the indole pulse caused *E. coli* BW25113 to enter into the stationary phase earlier and showed higher viability in the long stationary phase than Δ*tnaA* mutant. On another note, despite the biological relevance of the high concentration of indole on *E. coli*, less attention was given because it required indole concentration 20-fold higher than normally occur in LB medium.

To verify the effect of indole on cell growth, OD of cultures supplemented with exogenous indole in the range of 0–5 mM was analyzed. Figure 5d shows that the OD of culture without the addition of indole reached 1.15 ± 0.0059 at 24 h incubation, whereas the culture with 0.5 and 1 mM exogenous indole showed a slight reduction in growth, reaching 1.079 ± 0.0098 and 0.908 ± 0.0078, respectively. Moreover, the culture with a high concentration of exogenous indole of 5 mM showed no growth during incubation (0.091 ± 0.0077), confirming the effect of indole on cell growth of *E. coli.*

## Conclusion

The metabolic compound indole produced by bacteria can exert several biological functions including plasmid stability, virulence control, and biofilm formation during cell growth. In the current study, a SERS method was developed to quantitatively detect the production of indole by *E*. *coli* in LB media. This method was able to detect indole as low as 0.0886 mM in standard solution, which was lower than the biologically relevant concentration of indole that *E. coli* produces. The study also revealed that in the presence of 10 mM exogenous tryptophan the CFU/mL in the media at the end of the incubation period showed a three-log reduction in comparison with the cells grown in media without supplementation. Therefore, the proposed method could be applied as a metabolic product fingerprinting tool for analyzing microbial community. Future studies could extend the use of the method to study the intracellular spatial distribution of bioactive metabolites in cells during the different growth phases and chemical interactions between different species.
